# Metagenomic next-generation sequencing promotes pathogen detection over culture in joint infections with previous antibiotic exposure

**DOI:** 10.3389/fcimb.2024.1388765

**Published:** 2024-08-26

**Authors:** Zongyan Gao, Wendi Zheng, Meng Zhang, Yanhua Gao, Jincheng Huang, Xiao Chen, Zhipeng Dai, Zhenyu Song, Jiawei Feng, Qianqian Cao, Yi Jin

**Affiliations:** ^1^ Department of Orthopedics, Henan Provincial People’s Hospital, Zhengzhou, China; ^2^ Department of Anesthesia, People’s Hospital of Anyang City, Anyang, China

**Keywords:** metagenomic next-generation sequencing, joint infection, antibiotics, culture, pathogen

## Abstract

**Objective:**

To investigate the diagnostic value of metagenomic next-generation sequencing (mNGS) in detecting pathogens from joint infection (JI) synovial fluid (SF) samples with previous antibiotic exposure.

**Methods:**

From January 2019 to January 2022, 59 cases with suspected JI were enrolled. All cases had antibiotic exposure within 2 weeks before sample collection. mNGS and conventional culture were performed on SF samples. JI was diagnosed based on history and clinical symptoms in conjunction with MSIS criteria. The diagnostic values, including sensitivity, specificity, positive/negative predictive values (PPV/NPV), and accuracy, were in comparison with mNGS and culture.

**Results:**

There were 47 of the 59 cases diagnosed with JI, while the remaining 12 were diagnosed with non-infectious diseases. The sensitivity of mNGS was 68.1%, which was significantly higher than that of culture (25.5%, *p*<0.01). The accuracy of mNGS was significantly higher at 71.2% compared to the culture at 39.0% (*p <*0.01). Eleven pathogenic strains were detected by mNGS but not by microbiological culture, which included *Staphylococcus lugdunensis*, *Staphylococcus cohnii*, *Finegoldia magna*, *Enterococcus faecalis*, *Staphylococcus saprophytics*, *Escherichia coli*, *Salmonella enterica*, *Pseudomonas aeruginosa*, *Acinetobacter pittii*, *Brucella ovis*, and*Coxiella burnetii.* Antibiotic therapy was adjusted based on the mNGS results in 32 (68.1%) patients, including 12 (25.5%) and 20 (42.6%) patients, in whom treatment was upgraded and changed, respectively. All JI patients underwent surgery and received subsequent antibiotic therapy. They were followed up for an average of 23 months (20-27 months), and the success rate of treatment was 89.4%. Out of the 33 patients who had positive results for pathogens, reoperation was performed in 1 case (3.03%), while out of the 14 cases with negative results for both mNGS and cultures, reoperation was performed in 4 cases (28.6%).

**Conclusions:**

mNGS has advantages over conventional culture in detecting pathogens in SF samples from JI patients previously treated with antibiotics, potentially improving clinical outcomes.

## Introduction

Joint infections (JI) include septic arthritis (SA) and prosthetic joint infections (PJI), which can be caused by bacteria or fungi that enter the joint through the bloodstream or from a nearby infection. JI is associated with acute bacteremia, joint destruction, chronic pain, permanent dysfunction, and even death (Yombi et al., 2017; Huang et al., 2020b). As the population grows and ages, the burden of JI, particularly PJI, on the healthcare system is becoming increasingly significant (Colston and Atkins, 2018).

A timely and accurate microbiological diagnosis is crucial for the treatment of JI (Osmon et al., 2013). Conventional culture is still the gold standard for identifying pathogenic microorganisms. Unfortunately, the incidence of culture positivity ranges from 40% to 70% and is affected by several factors, including previous antibiotic exposure, fastidious pathogens, and biofilm formation (Trampuz et al., 2007; Huang et al., 2020b). Additionally, the turnaround time of culture always takes 5-14 days or even longer. Due to the urgency of JI treatment and limited culture positivity, empirical antibiotic treatment prior to the identification of pathogenic microorganisms remains common. Previous antibiotic exposure is an important cause of negative cultures, which can result in a reduction of culture positivity to 7%-42% (Cai et al., 2020; Yu et al., 2023). In contrast, the reoperation rate for the treatment of culture-negative joint infections exceeds 50% (Tan et al., 2018), which can be four times higher than that of culture-positive cases (Mortazavi et al., 2011).

mNGS is a combination of high-throughput sequencing and bioinformatics analysis that directly detects nucleic acids extracted from samples. Then, it coordinates with a reference database to determine the species and abundance of all microorganisms in the sample within a short turnaround time (1-3 days) (Indelli et al., 2021). Studies found that mNGS has good diagnostic value for detecting JI pathogenic microorganisms. The sensitivity ranges from 80.6% to 95.45%, and the specificity ranges from 84.6% to 90.91%, which was significantly better than conventional culture (Cai et al., 2020; Yu et al., 2023). In addition, mNGS showed good diagnostic values for different sample types. For pathogen detection in JI, synovial fluid (SF) has equal diagnostic value to tissue and sonicate fluid (Huang et al., 2020a).

For patients with previous antibiotic exposure, mNGS has been shown to be superior to traditional culture methods for detecting pathogenic microorganisms in cases of infective endocarditis, solid organ transplantation (Everhart and Henshaw, 2023), and aspiration pneumonia (Liang et al., 2022). However, studies on the diagnostic value of mNGS for JI cases with previous antibiotic exposure are limited. A controlled study based on intraoperative tissue specimens from 13 patients with PJI showed that mNGS has higher sensitivity than culture (Yu et al., 2023). Another study evaluated the performance of mNGS and culture from different types of samples, including SF, tissue specimens, and sonicate fluid from PJI patients, and found that mNGS was less affected by previous antibiotic exposure (Huang et al., 2020b). The diagnosis of the pathogenesis based on SF, which can be easily obtained preoperatively, is an important guide for selecting intraoperative antibiotics.

Here, in cases with previous antibiotic exposure, we evaluated the clinical value of mNGS and culture in detecting pathogens in SF of JI.

## Patients and methods

### Study population

This study was a retrospective analysis of patients with suspected JI who eventually underwent surgical treatment in our department from January 2019 to January 2022. Patients with JI are diagnosed by physicians and orthopedic surgeons based on history, clinical manifestations, laboratory tests, and imaging. Consent was obtained from the partaker to receive preoperative arthrocentesis to obtain joint fluid for mNGS testing. This study was reviewed and approved by the Henan Provincial People’s Hospital Institutional Review Board (202081).

### Definitions

The JI cases in this study included SA and PJI. Following our protocol, we diagnose SA in patients with a history of a warm, swollen, and tender joint. We perform arthrocentesis to obtain SF samples for microbiological analyses. PJI was defined with the criteria of the Musculoskeletal Infection Society (MSIS) (Parvizi et al., 2018).

The inclusion criteria were as follows: (1) diagnosis confirmation through preoperative and intraoperative tests, (2) patients had received antibiotics within 2 weeks prior to collecting the SF sample, and (3) perioperative and follow-up data were fully recorded. The exclusion criteria were as follows: (1) diagnosis is indeterminate due to insufficient clinical and laboratory evidence; (2) samples were unqualified, such as contamination and inadequate volume for tests.

### Clinical sample collection and processing

SF samples were collected by needle aspiration preoperatively. Samples were immediately separated into aerobic and anaerobic blood culture bottles (BD Biosciences, Sparks, USA) for microbiology culture, a free sterile container for mNGS, and an ethylenediaminetetraacetic acid (EDTA) vial for cytological analysis. Ultrasound-guided puncture was performed for the hip joint. During surgery, tissue specimens were collected from the most inflammatory site. After microbiology culture samples were collected, they were sent to the laboratory within 1 h. For mNGS tests, samples were stored at -20°C and sent to the molecular lab of the biological company (Practice Medicine Company, Zhengzhou) within 24 h.

### Conventional microbiology culture

The samples were homogenized, and routine cultures were simultaneously performed, including aerobic and anaerobic bacterial cultures, fungal cultures, and acid-fast bacilli cultures. SF (≥1 mL) and tissue samples were placed in sterile tubes, and 3 mL of brain heart infusion broth was added, vortexed and shaken at 2500 rpm/min for 15 minutes in a vortex oscillator (Thermo Fisher Scientific, USA) after sealing. Then tissue samples were placed in a fully automatic rapid grinding instrument (Jingxin Industrial Development, Shanghai, China) at 40 Hz for 60-90 seconds. The obtained homogenate was inoculated on a blood agar plate (Thermo Fisher Scientific, USA) for microbial culture under anaerobic and aerobic conditions. One milliliter of homogenate was injected into Bactec Plus/F aerobic culture vials (BD Biosciences, Sparks, MD, USA) and Bactec Lytic/10/F anaerobic culture vials (BD Biosciences, Sparks, MD, USA). The incubation time was extended up to 2 weeks. All bacteria or fungi isolated were identified using the Vitek II system (Biomerieux, USA) and matrix-assisted laser desorption ionization-time of flight mass spectrometry, using a Bruker Daltonics system (Billerica, MA).

### Metagenomic next-generation sequencing and analysis

#### Sample processing and sequencing

Following the collection of SF, 2 mL of the sample was immediately removed and inactivated at 80°C for 10 minutes. A 1.5 mL microcentrifuge tube containing 0.8 mL of the sample and 2 g of 0.5 mm glass beads was placed on a mixing platform at a speed of 3000 RPM for 15 minutes. Then, all samples were centrifuged at 13000 RPM for 10 minutes. Six hundred microliters of the sample was transferred into a 2.0 mL microcentrifuge tube, and the DNA was extracted using the TIANamp Micro DNA Kit (DP316, Tiangen Biotech Co., Ltd.) according to the recommended protocol in the instructions. According to the protocol of the BGISEQ-200 sequencing platform, the DNA library was constructed by DNA fragmentation, end-repair, adapter-ligation, and PCR amplification. The constructed library was qualified by Agilent 2100 (Agilent Technologies, USA) and Qubit 4.0 (Thermo Fisher, USA). The qualified double-strand DNA library was transformed into a single-stranded circular DNA library through DNA denaturation and circularization. DNA nanoballs (DNBs) were generated from single-stranded circular DNA using rolling circle amplification (RCA). The DNBs were qualified using Qubit 4.0. Qualified DNBs were loaded into the flow cell and sequenced (50 bp, single-end) on the BGISEQ-200 platform.

#### Bioinformatic analysis

High-quality sequencing data were generated by removing low-quality and short (length < 35 bp) reads using fastp software (Chen et al., 2018) followed by computational subtraction of human host sequences mapped to the human reference genome (hg38) using STAR (Dobin et al., 2013) alignment. After the removal of low complexity and duplicated reads using PRINSEQ algorithms (Schmieder and Edwards, 2011), the remaining data were classified using Kraken2 software (Wood et al., 2019, p. 2) by a pathogen reference database that was gathered from multiple public genome databases, including FDA-ARGOS (Food & Drug Administration- dAtabase for Reference Grade micrObial Sequences), BV-BRC (Bacterial and Viral Bioinformatics Resource Center, version 3.28.9), EuPathDB (Eukaryotic Pathogen Genome Database, Release 52), and NCBI GenBank (National Center for Biotechnology Information, Release 242). The pathogen reference database comprises 28,516 bacterial taxa, 8,046 viral taxa, 2,076 fungal taxa, and 429 parasite taxa. The sequencing data list was analyzed in terms of species-specific read number (SSRN), reads per million(RPM), and genome coverage (%).

The pathogens detected by mNGS were considered positive according to the previously reported threshold as follows (Miller et al., 2019; Hao et al., 2023). (I) Bacteria (excluding mycobacteria): Organisms with a coverage rate of at least 10 times that of other organisms were deemed to be pathogenic species. For an organism that did not match the negative control pathogen, it was the pathogenic species if the number of reads mapped to the pathogen at the genus level was ≥10. For an organism that matched the negative control pathogen, it was considered positive if the coverage rate was ≥2% and the number of reads strictly mapped to pathogen at the genus level was ≥10 in two consecutive tests. (II) Mycobacterium: If the number of reads strictly mapped to a pathogen at genus level ≥1 and the number was in the top 10, it was a pathogenic species. (III) Fungi: If the number of reads mapped to pathogens at genus or species level ≥10 and were in the top 10 for bacteria, they were considered positive.

### Management and follow-up

**Figure 1:**
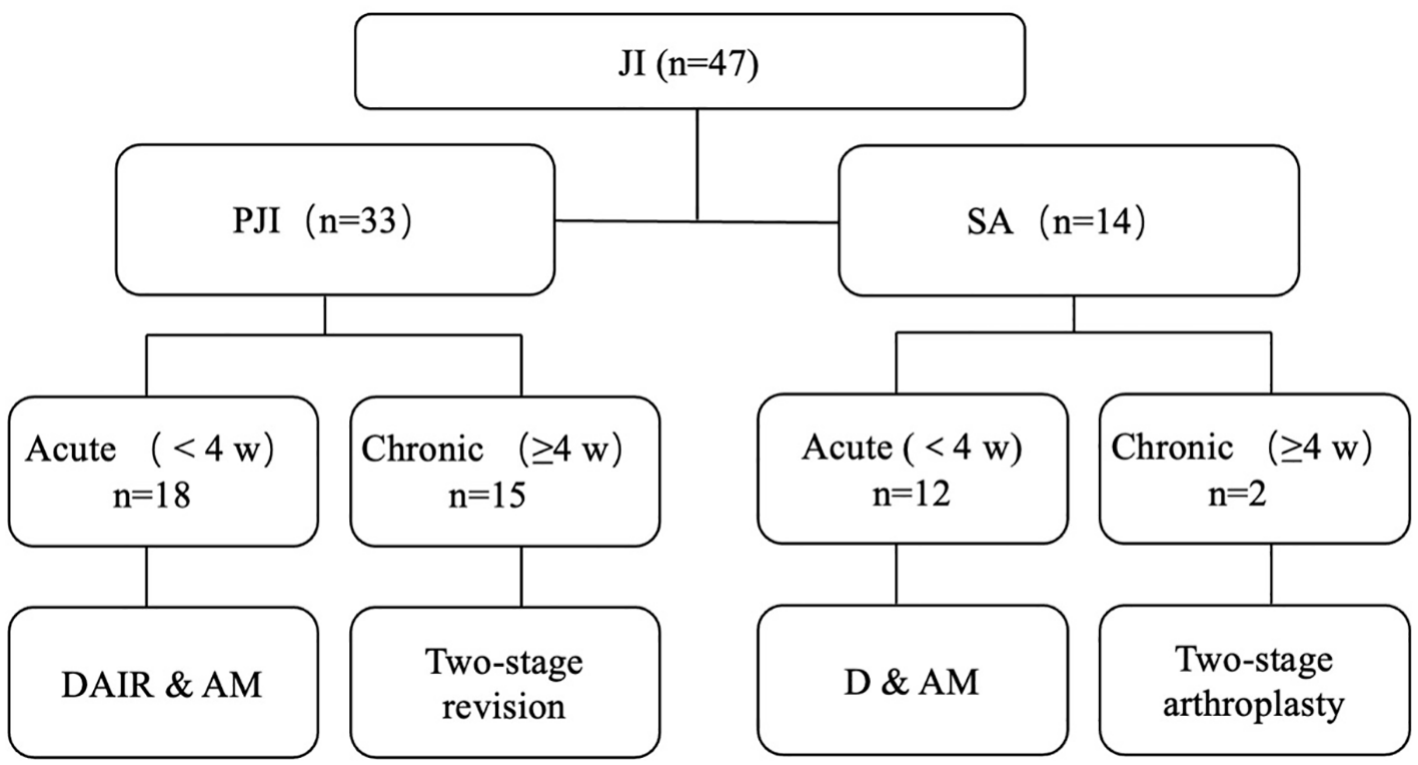
Management strategy. JI, joint infection; PJI, periprosthetic joint infection; SA, primary septic arthritis; DAIR, debridement and retention of the implant; AM, antibiotic management; D & AM, debridement and antibiotic management.

Management strategy (**Figure 1**): The classification of JI into acute (<4 weeks) and chronic (≥4 weeks) is based on the onset time. Chronic JI was typically treated using a two-stage revision/arthroplasty procedure: (1) surgical debridement (and implant removal in PJI) is followed by implantation of a spacer made of polymethyl methacrylate mixed with antibiotics. Intra- and postoperative antibiotic selection was determined by orthopedic surgeons and infection physicians based on preoperative pathogen diagnosis. If pathogenetic testing was negative, empirical antibiotic therapy was administered. (2) Three months later, a revision arthroplasty was performed after confirming the cure of the infection through clinical symptoms and laboratory examinations. Outpatient follow-up was conducted at 3 months, 6 months, and every 6 months thereafter.

### Statistical analysis

SPSS 26.0 (SPSS Inc., Chicago, Illinois, USA) was used in this study. The independent samples t-test was used to compare groups of normally distributed data; the Mann-Whitney U test was used to compare groups of non-normally distributed data. Comparisons of sensitivity, specificity, positive predictive value, negative predictive value, and accuracy were performed using the chi-square test, and related 95% confidence intervals (CIs) in culture and NGS techniques. We performed an *a priori* power analysis to determine the minimum sample size necessary to achieve statistical significance. Using previously published data (Huang et al., 2020a), we estimated the sensitivity of microbiological culture at 61.2% and mNGS at 95.9%. We determined that a sample size of 36 patients would achieve a power of 80.0% with an alpha error of 0.05. A *p*-value < 0.05 was considered significant.

## Results

### Sample characteristics

**Figure 2:**
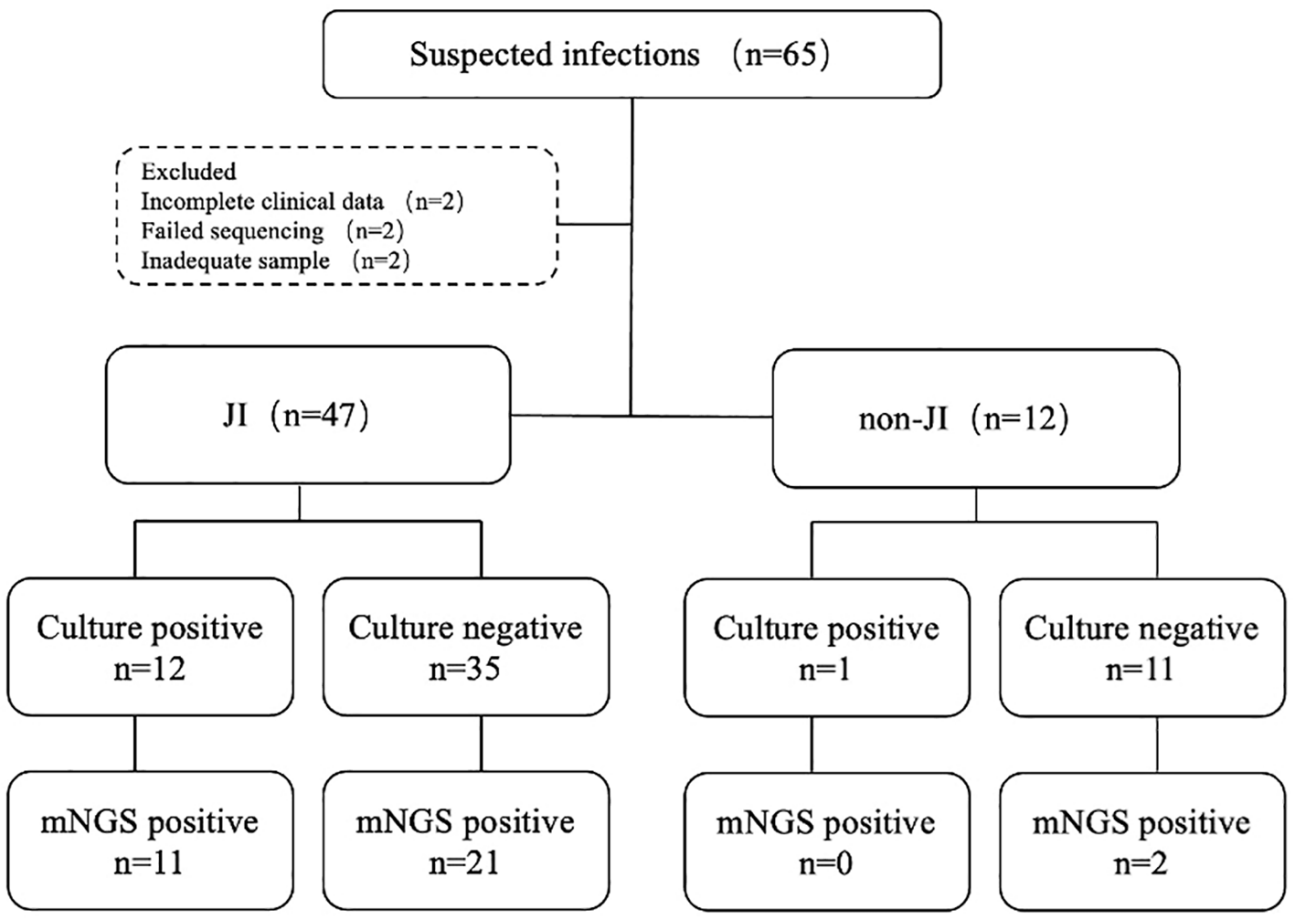
Flowchart detailing enrolment and microbiological results of study samples. JI, joint infection.

**Figure 3:**
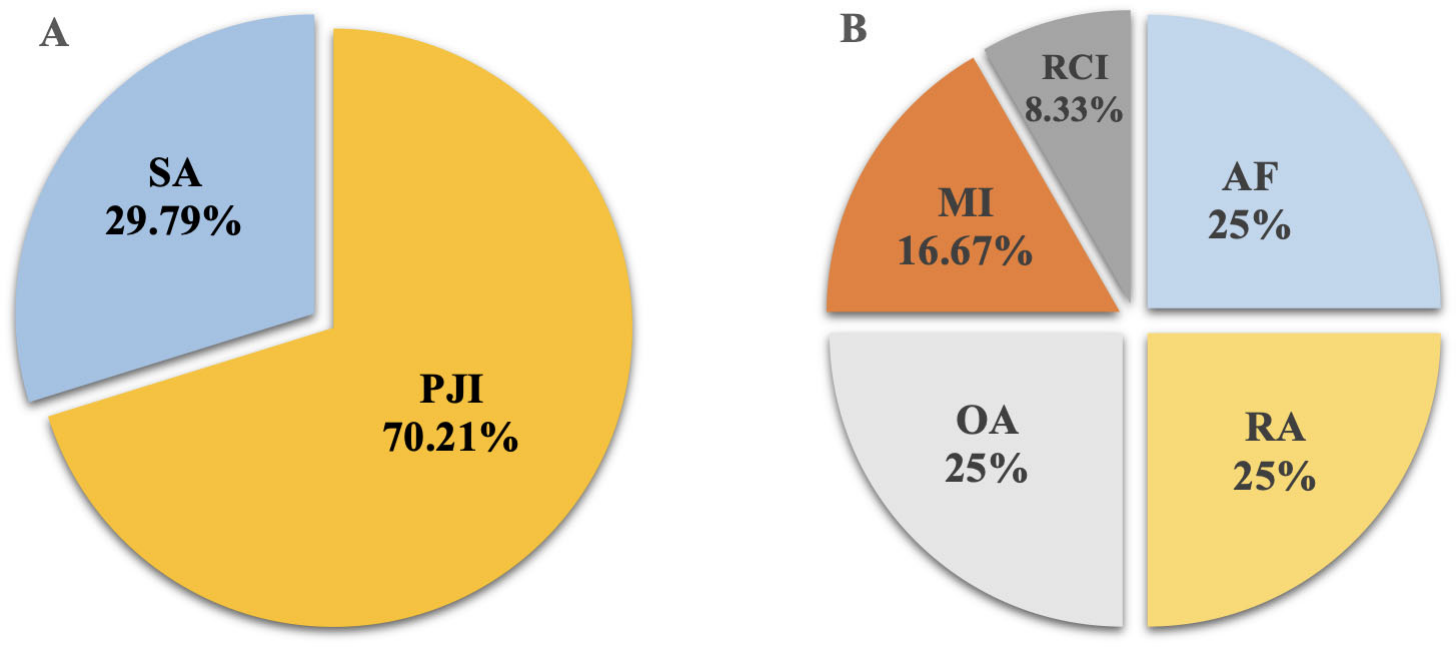
The distribution of JI and non-JI. **(A)** JI group; **(B)** non-JI group. SA, septic arthritis; PJI, periprosthetic joint infection; OA, osteoarthritis; RA, rheumatoid arthritis; AF, aseptic failure; MI, meniscus injury; ROI, rotator cuff injury.

This study retrospectively analyzed 65 cases with suspected JI. Six cases were excluded due to various reasons, including failed sequencing, incomplete clinical data, and inadequate volume of sample for culture and mNGS (**Figure 2**). Fifty-nine cases were enrolled, including 47 cases confirmed with JI, and 12 cases confirmed with non-JI. The distribution of JI and non-JI is shown in **Figure 3**. Clinical data are listed in **Table 1**. Among the 47 cases with a clinical diagnosis of JI, the time interval between antibiotic discontinuation and sample collection was 6.73 ± 2.69 days, 4.67 ± 2.03 days, 3.67 ± 1.63 days, and 5.67 ± 1.76 days in the mNGS and culture both-positive group, the mNGS-positive group, the culture-positive group, and the both-negative group with mNGS and culture, respectively. The time interval of the culture-positive group was found to be significantly shorter than that of the both-positive group (*p*=0.027).

**Table 1:** Clinical and laboratory characteristics of patients.

Characteristics	JI(n=47)	non-JI(n=12)	p-value
Mean age, yrs, mean (SD)	61.4 (12.8)	46.5 (19.9)	0.002^a^
Sex, n (%			
** **Male	25 (53.2)	5 (41.7)	0.476^b^
** **Female	22 (46.8)	7 (58.3)	
Site of infection, n (%)			
** **Knee	28 (59.6)	7 (58.3)	
** **Hip	18 (38.3)	4 (33.3)	
** **Shoulder	1 (2.13)	1 (8.33)	
Laboratory findings			
** **NEUT, ×10^9^/L, mean (SD)	6.25 ± 2.58	3.71 ± 1.68	0.002^a^
** **NEUT%, median (Q1, Q3)	66.1 (49.2, 78.9)	50.2 (41.2, 59.0)	0.069^c^
** **CRP, mg/L, median (Q1, Q3)	31.2 (21.2, 55.8)	9.05 (6.44, 20.6)	<0.001^c^
** **ESR, mm/h, median (Q1, Q3)	49 (39,77)	26 (18.5, 45)	0.005^c^
** **SF-PMN, ×10^6^/L, median (Q1, Q3)	9130 (8045.5, 10793.5)	2065.5 (834, 2965)	<0.001^c^
** **SF-PMN%, median (Q1, Q3)	89 (81, 91)	40.5(34, 50)	<0.001^c^
Infection duration, weeks, median (Q1, Q3)	7 (3,10)	–	–
Follow-up time, months, mean (SD)	23.7 (4.52)	12.3 (5.69)	<0.001^a^

NEUT, serum neutrophil count; NEUT, serum neutrophil percent; CRP, serum c-reactive protein; ESR, serum erythrocyte sedimentation rate; SF-PMN, synovial fluid polymorphonuclear count; SF-PMN%, synovial fluid polymorphonuclear percent. a: Independent-samples t-test, data are described as mean (standard deviation, SD); b: Chi-squared test; c: Mann-Whitney U test, data are described as medium (first quartile, third quartile).

### Concordance between the mNGS and culture

**Figure 4:**
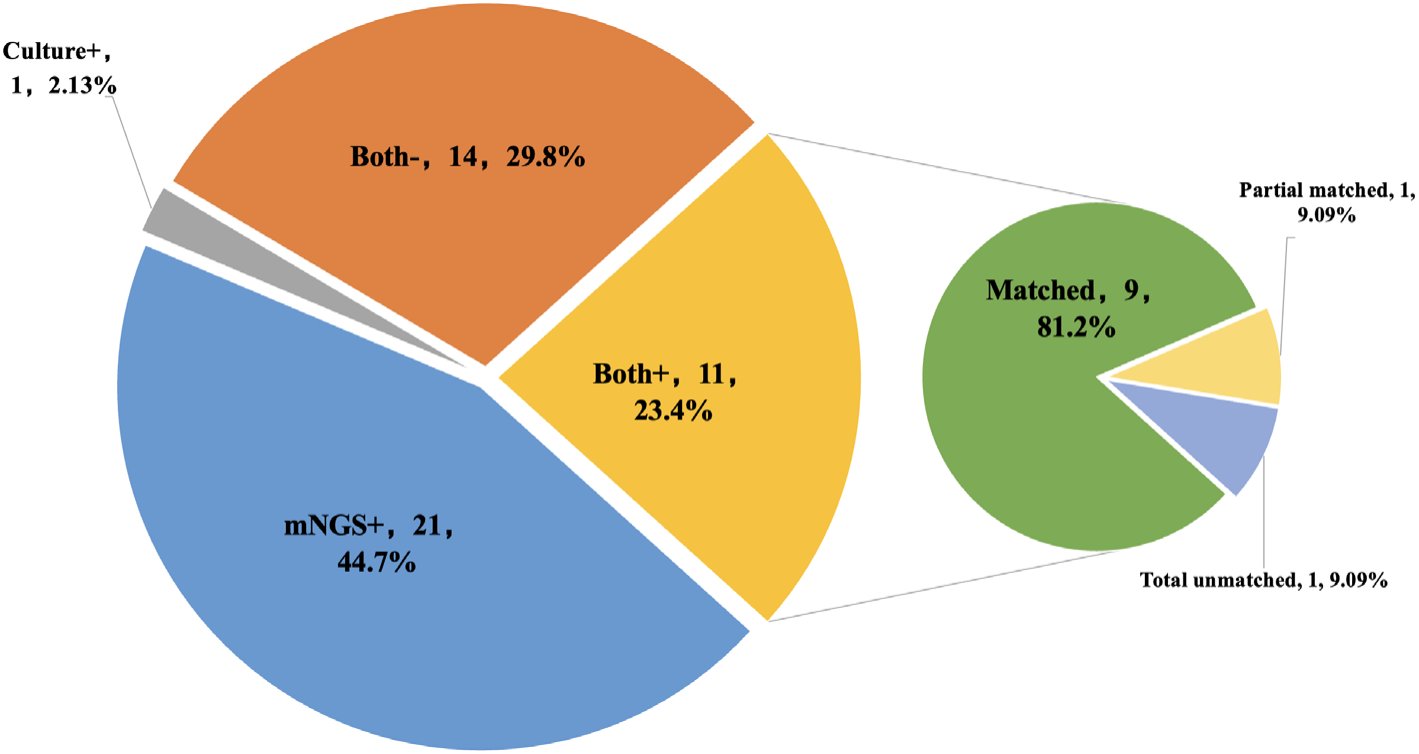
The concordance of results between metagenomic next-generation sequencing (mNGS) and culture. Both+, results of mNGS and culture were both positive; Both-, results of mNGS and culture were both negative; mNGS+, only the mNGS result was positive, culture was not; Culture+, only the culture result was positive, mNGS was not.

mNGS and cultures were both positive in 11 of 47 (23.4%) samples and were both negative in 14 (29.8%) samples. Twenty-one samples were positive by mNGS only (44.7%), and 1 was positive by culture only (2.13%).Among the 11 samples that were positive for both mNGS and culture, 9 (81.2%) had fully matching results; one case (9.09%) had partially matched results, *Staphylococcus aureus* was detected by both methods while *Pseudomonas* was missed by mNGS; one case (9.09%) had total unmatched results, *Finegoldia magna* was detected by mNGS while *Staphylococcus hominis* was detected by culture (**Figure 4**).

Eleven pathogenic strains were detected by mNGS but not by microbiological culture, which included *Staphylococcus lugdunensis*, *Staphylococcus cohnii*, *Finegoldia magna*, *Enterococcus faecalis*, *Staphylococcus saprophytics*, *Escherichia coli*, *Salmonella enterica*, *Pseudomonas aeruginosa*, *Acinetobacter pittii*, *Brucella ovis*, and *Coxiella burnetii* (**Table 2**).

**Table 2:** Pathogens detected by mNGS and culture.

Pathogens	mNGS	Culture
Gram-positive cocci		
*Staphylococcus aureus*	7	5
*Staphylococcus epidermidis*	4	3
*Staphylococcus haemolyticus*	1	1
*Staphylococcus lugdunensis*	2	0
*Staphylococcus hominis*	1	1
*Staphylococcus cohnii*	1	0
*Finegoldia magna*	2	0
*Enterococcus faecalis*	1	0
*Staphylococcus saprophytics*	1	0
Gram-negative bacillus		
*Stenotrophomonas maltophilia*	1	1
*Klebsiella pneumoniae*	2	1
*Escherichia coli*	4	0
*Salmonella enterica*	1	0
*Pseudomonas stutzeri*	0	1
*Pseudomonas aeruginosa*	1	0
*Acinetobacter pittii*	1	0
*Brucella ovis*	1	0
*Fungi*	4	2
*Coxiella burnetii*	1	0
Total	34	14

### Diagnostic and clinical values of mNGS and culture

The mNGS assay yielded a sensitivity of 68.1% (52.7-80.5%), which was significantly higher than 25.5% of culture (*p* < 0.01). The accuracy of mNGS was 71.2%, which was significantly higher than 39% of culture (*p* < 0.01). While there were no statistically significant differences in specificity, PPV, and NPV (**Table 3**). The turnaround time for mNGS was 1 day, significantly shorter than 5 days for culture (*p* < 0.01).

**Table 3:** Comparison of diagnostic values among mNGS and culture.

Methods	Sensitivity %(95%Cl)	Specificity %(95%Cl)	PPV%(95%Cl)	NPV %(95%Cl)	Accuracy %(95%Cl)	Turnaround Time(days), (95%Cl)
Culture	25.5 (14.4, 40.6)	91.7 (59.8, 99.6)	92.3 (62.1, 99.6)	23.9 (13.1, 39.1)	39.0 (38.2, 39.0)	5.0 (4.0, 5.0)
mNGS	68.1 (52.7, 80.5)	83.3 (50.9, 97.1)	94.1 (78.9, 99.0)	40.0 (21.8, 61.1)	71.2 (70.5, 71.9)	1.0 (1.0, 1.0)
p-value	< 0.01	1.00	0.66	0.156	< 0.01	< 0.01

PPV, positive predictive value; NPV, negative predictive value. Cl, confidential interval.

Antibiotic treatment was adjusted before operations based on the mNGS results in 32 (68.1%) patients, including 12 (25.5%) and 20 (42.6%) patients, in whom treatment was upgraded, and changed, respectively.

Five JI patients required reoperation, and all were diagnosed with PJI of the knee. Of these, one PJI patient initially received prosthesis removal and spacer implant. The preoperative SF mNGS and culture tests took one and five days, respectively, and were both negative. Vancomycin was empirically chosen for local treatment in the spacer. Two weeks later, the culture-based preoperative sample reported *Staphylococcus epidermidis* and *Candida parapsilosis*. Thus, fluconazole was used for postoperative management. After 5 months, the recurrence of surgical site infection occurred. The debridement was performed, and the spacer impregnated with fluconazole and vancomycin was implanted. Oral fluconazole was continued for 1 year following the surgery. With 2 years of follow-up, no recurrence of infection was observed. The other 4 cases were negative for both mNGS and culture before operation and received debridement and retention of the implant (DAIR) with following empirical antibiotic management. The infection recurred 4 to 14 weeks after surgery. Then two-stage revisions were performed. With over 21 months of follow-up, no recurrence of infections was observed. In summary, among 33 JI patients with positive results of pathogens, 1 case (3.03%) required reoperation, while among 14 cases with negative results of pathogens, 4 cases (28.57%) required reoperation.

## Discussion

In the present study, the performances of mNGS and conventional culture in detecting pathogens from SF samples in JI patients with previous antibiotic exposure were systematically compared. Traditional cultures depend on the growth and multiplication of pathogens. However, the presence of antibiotics can interfere with this process, inhibiting pathogen growth and potentially leading to false-negative results. In contrast, the mNGS involves direct sequencing of the nucleic acids of infectious pathogens present in joint fluid, bypassing the need for pathogen cultivation. Consequently, even when pathogens are suppressed by antibiotics, mNGS can enhance the detection rate, increasing the likelihood of obtaining positive results (Benamu et al., 2022; Feng et al., 2024). The results showed that mNGS has higher sensitivity and accuracy than culture and can detect more potential pathogens from a single SF sample. Moreover, it took only 1 day for mNGS to generate a final report, which could provide prompt guidance for targeted antimicrobial treatment during emergency surgery in clinical practice compared to culture.

This study has shown that mNGS offers several advantages over conventional culture when it comes to diagnosing JI using SF samples. First, the sensitivity and accuracy of mNGS were less affected by antibiotic treatment. This result was consistent with prior literature. mNGS is less affected by prior antibiotic exposure than culture in the detection of pathogens in a variety of diseases, including infective endocarditis (Eichenberger et al., 2023), tissue transplantation (Everhart and Henshaw, 2023), and lung and central nervous system infections (Miao et al., 2018). Nucleic acids serve as the foundation for mNGS diagnostics, and the half-life of cell-free nucleic acids in synovial fluid remains under-researched. However, due to the slower metabolism of synovial fluid compared to blood, studies have found that in animal models of arthritis, the concentration of cell-free nucleic acids in synovial fluid is more than twice that in bloodstream (Panizzi et al., 2023). Consequently, the utilization of SF samples for mNGS detection is theoretically more sensitive and accurate than the use of blood samples. For the pathogenic detection of JI, the positive rate of mNGS using tissue specimens was 69.5%, significantly higher than culture with 23.1% (Yu et al., 2023); comprehensive analysis of SF, sonicate fluid, and tissue samples and demonstrated that the sensitivity of mNGS was 89.7%, which was significantly higher than that of culture at 61.5% (Huang et al., 2020b). Second, mNGS detected more species of pathogens than culture did from a single SF sample. Eleven pathogens were detected only by mNGS in this study, including *Staphylococcus lugdunensis*, *Staphylococcus cohnii*, *Finegoldia magna*, *Enterococcus faecalis*, *Staphylococcus saprophytics*, *Escherichia coli*, *Salmonella enterica*, *Pseudomonas aeruginosa*, *Acinetobacter pittii*, *Brucella ovis*, and *Coxiella burnetii.* In addition, mNGS exhibits higher sensitivity in detecting fungi (**Table 2**). Since the detection of mNGS is based on sequences of genes of pathogens rather than culture based on live bacteria, mNGS has a higher sensitivity than culture for some fastidious bacteria (Wang et al., 2020; Everhart and Henshaw, 2023), fungi (Huang et al., 2020a; Liang et al., 2022), and mycobacterium (Ko et al., 2019). Third, the turnaround time for mNGS was one day, which is significantly faster than culture, which took 4-14 days. For JI patients requiring urgent treatment, a shorter turnaround time is crucial in guiding antibiotic use (Benamu et al., 2022; Feng et al., 2024), especially in the selection of intraoperative local antibiotics.

Based on the above advantages, 32 out of 47 JI patients (68.1%) were given antibiotic adjustment based on mNGS results in this study. In 12 cases (25.5%), antibiotics were upgraded, and in 20 cases (42.6%), inappropriate empiric use of antibiotics was identified and changed. After a mean follow-up of 23.7 months, the treatment success rate was 89.4%, which is higher than the 67-71.6% reported in the literature (Corona et al., 2020; Burr et al., 2022).

In this study, five JI patients required reoperation. One of these patients only had a positive pathogenic result with culture, but the report came 2 weeks after the operation. The pathogens were identified as *Staphylococcus epidermidis* and *Candida parapsilosis*. However, the implanted spacer was not impregnated with antifungal agents. As a result, the infection recurred after 5 months. The debridement and implant of a spacer impregnated with antifungal agent were performed. After a 2-year follow-up, there was no recurrence of infection. Thus, timely pathogen identification is crucial. The other 4 cases were negative for both mNGS and culture. Among cases with both negative results, the reoperation rate was 28.6%, which is lower than the 50% reported in the literature (Tan et al., 2018), but still significantly higher than the group with positive pathogen testing.

It is not sufficient to rely on mNGS results alone to diagnose fungal osteoarticular infections, due to the possibility of contamination and false positives. In this study, a total of 4 fungal infections were diagnosed by mNGS, with one of these cases subsequently validated by culture results. The remaining 3 patients were clinically confirmed to have chronic joint infections (more than 16 weeks), which were negative in multiple SF and tissue cultures conducted before and after the mNGS examination. These patients were treated with a variety of empirical antibiotics, excluding antifungal drugs; however, the outcomes were not favorable. Following the mNGS results, which indicated a fungal infection, we consulted with infection specialists and proceeded with debridement and antifungal therapy. After a period of more than six months of postoperative antifungal therapy and over 20 months of follow-up, the infection was well controlled, leading to the clinical diagnosis of fungal joint infection. The positive rate of fungal culture and histological examination is relatively low. Currently, the diagnosis and treatment based on mNGS have been supported by several clinical studies (Li, 2022; Wang et al., 2022).

Previous antibiotic exposure affected the diagnostic value of mNGS on JI. In this study, the antibiotics empirically used and the results of pathogenic detections are shown in **Table 4**. The rate of negative results for both mNGS and culture was highest in people who had previously taken vancomycin (66.7%) and carbapenems (100%), followed by macrolides (33.3%) and combination antibiotic treatment (33.3%). The combinations included first- to third-generation cephalosporins, β-lactams, and quinolones. Therefore, upgrading antibiotics in empiric therapy needs to be prudent, and obtaining samples in advance is highly recommended.

**Table 4:** Previously used antibiotics and pathogen results.

Antibiotics	Both+	Culture+	mNGS+	Both- (%)
1^st^ & 2^nd^ cephalosporins (or/iv)	3		7	2 (16.7%)
3^rd^ cephalosporins (iv)	1		2	0
β-lactams (or)	3		1	1 (20.00%)
Quinolones (or/iv)		1	3	1 (25.00%)
Macrolides (or)	2		4	3 (33.33%)
≥2 (or/iv)	2		2	2 (33.3%)
Vancomycin (iv)			2	4 (66.7%)
Carbapenems (iv)				1 (100%)

Both+, results of mNGS and culture were both positive; Both-, results of mNGS and culture were both negative, % rate of cases with Both- in which this class of antibiotic was used; mNGS+, only the mNGS result was positive, culture was not; Culture+, only the culture result was positive, mNGS was not; 1^st^, 2^nd^, 3^rd^ cephalosporins, first, second, and third generation cephalosporins; ≥2, combination antibiotic treatment; Or, oral; Iv, intravenous.

There were still some limitations in this study: (1) This study was conducted at a single center and included a limited number of patients, which may affect the reliability of the conclusions. To strengthen the results, multicenter studies should be conducted in the future; (2) The diagnostic accuracy of mNGS compared to culture would be affected due to the lack of non-infection samples, as mNGS is a highly sensitive method that is sensitive to contamination. (3) As a retrospective study, the findings of this research may be influenced by selection bias. For instance, previous antimicrobial treatment was more common in PJIs caused by acute infection, particularly by highly virulent pathogens. In these patients, antimicrobial therapy has a smaller effect on culture positive in these patients than in those with chronic infection (Hao et al., 2023). (4) Analyzing the effects of antibiotics on mNGS based on limited samples is challenging due to the diversity of empirical antibiotics in terms of classes, administration routes, and treatment durations. In the future, with the increase in sample size and the maintenance of more detailed records, more comprehensive research is expected to be conducted in this area.

In conclusion, based on a single SF sample, mNGS was found to have significantly higher sensitivity and accuracy than conventional culture for JI patients with previous antibiotic exposure. Furthermore, mNGS was able to diagnose more pathogenic microorganisms in a shorter turnaround time, which can guide antibiotic treatments more effectively and potentially improve clinical outcomes.

## Data Availability

All data have been uploaded into EMBL database with accession number is PRJEB72431.
